# Impact of sample refrigeration and freezing on the bacteriological counts of different bedding materials for dairy cows

**DOI:** 10.1186/s12917-024-04247-w

**Published:** 2024-09-12

**Authors:** Sara Fusar Poli, Valentina Monistero, Claudia Pollera, Gustavo Freu, Valerio Bronzo, Renata Piccinini, Marco Nocetti, Giulia Sala, Marcos Veiga dos Santos, Paolo Moroni, M. Filippa Addis

**Affiliations:** 1 Department of Veterinary Medicine and Animal Sciences, University of Milan, Lodi, Italy; 2 Laboratory of Animal Infectious Diseases (MiLAB), University of Milan, Lodi, Italy; 3 Department of Animal Nutrition and Production, School of Veterinary Medicine and Animal Science, University of São Paulo, Pirassununga, Brazil; 4 Consorzio del Formaggio Parmigiano Reggiano, Reggio Emilia, Italy; 5 Department of Veterinary Sciences, University of Pisa, Pisa, Italy; 6 Animal Health Diagnostic Center, Quality Milk Production Services (QMPS), Cornell University, Ithaca, NY USA

**Keywords:** Separated raw manure solids, Anaerobically digested manure solids, New sand, Bedding bacteriology

## Abstract

**Background:**

Different organic and inorganic bedding materials can be used in dairy farms. Among organic materials, there is an increasing interest in alternative substrates based on recycled manure solids (**RMS**). Microbiological analyses are crucial to monitor the microbial load and evaluate the presence of pathogens impacting animal welfare and health. However, logistic factors may hamper the possibility of immediately sending fresh samples to the laboratory, requiring storage in cooled conditions before analysis.

**Methods:**

We assessed the impact of sample refrigeration and freezing of different organic and inorganic bedding substrates including separated raw manure solids (**SRMS**), anaerobically digested manure solids (**ADMS**), and new sand (**NS**), on the total bacterial count (**TBC**) and on different microbial classes.

**Results:**

The TBC was higher in fresh NS and ADMS than in refrigerated and frozen samples of the same substrates; in addition, the TBC of ADMS was higher in refrigerated than frozen samples. The TBC of SRMS did not change significantly with refrigeration and freezing. Freezing reduced the total Gram-negative bacterial count more than refrigeration in all substrates. In fresh NS, Gram-negatives were higher than in both refrigerated and frozen NS. *Escherichia coli* counts were significantly lower in frozen than in refrigerated SRMS. However, both refrigeration and freezing of ADMS resulted in no *E. coli* growth. The coliform counts were also lower in frozen than refrigerated NS and SRMS. Frozen NS and ADMS showed lower counts compared to refrigeration for Gram-negative bacteria other than *E. coli* and coliforms. On the other hand, cold storage did not significantly impact the streptococci and streptococcus-like organisms (**SSLO**) count of all evaluated bedding substrates.

**Conclusion:**

Refrigeration and freezing affect the bacteriological results of bedding substrates, with freezing generally leading to lower counts than refrigeration. Whenever possible, preference should be given to analyzing fresh bedding samples, however, when necessary, refrigeration would be recommended over freezing, while acknowledging that the measured bacterial load might underestimate the actual microbial content.

## Introduction

Different substrates can be used as bedding materials in dairy farms. An appropriate substrate is essential for animal welfare, productivity, and health. Bedding materials of different types and qualities should always ensure thermal comfort and softness, be durable, and provide sufficient friction to allow animals to get up and lie down without slipping [1]. Several factors should be considered when choosing the right bedding materials, including economic and environmental sustainability, dryness, bacterial growth inhibition, and compatibility with the manure-cleaning system [2].

The most widely used bedding materials can be categorized into two main groups: organic (e.g., straw, sawdust, hay, wood shavings, and compost) and inorganic (e.g., unused and recycled sand) [2, 3]. Each substrate has its advantages and disadvantages [4]. Organic substrates can better absorb moisture, are more compatible with manure disposal systems, are readily available in sufficient amounts, and are economically more sustainable [2]; however, it is important to consider that they generally have higher bacterial counts than inorganic materials. On the other hand, despite being inert and able to provide significant benefits such as a comfortable resting surface, a limiting effect on bacterial growth, and a cool surface to reduce heat stress [5], sand is not always readily available and compatible with manure management and disposal systems [2] due to its abrasive action [6].

Bedding supply, management, treatment, and manure disposal significantly impact the farm economy and the environment. As a results, dairy producers are increasingly interested in alternative, cost-effective, and easily available options, that can be advantageous for animals, humans, and the environment [7]. There is growing attention toward separated raw manure solids (**SRMS**) and anaerobically digested manure solids (**ADMS**) as bedding substrates. SRMS are the result of manure separation through screws or rollers into a solid fraction consisting mainly of undigested fibers and a liquid fraction. ADMS is the solid material resulting from the anaerobic digestion of manure and can represent a way to balance digester costs for biogas production [9, 10]. The use of SRMS and ADMS has been reported in the U.S., U.K., and The Netherlands [7]. These soft and non-abrasive materials can provide high cow comfort during lying time, low levels of hock lesions, and good udder hygiene [8], and constitute a virtuous management choice under sustainability and circular economy principles. Nevertheless, as a potential reservoir of microorganisms, it can increase the risk of intramammary infections (**IMI**). Therefore, it is necessary to monitor dry matter (DM) and pH and to control the moisture, which must be kept at optimum levels, when the material exits the separator. DM should be between 35% and 40% [8] to balance cow comfort and minimize microbial growth. If the moisture content exceeds this range, it can favor microbial proliferation, increasing the risk of infection. Proper monitoring and management of these parameters is critical to maintaining the health and well-being of the dairy herd.

In this scenario, microbiological analysis becomes important to monitor microbial growth and to keep cows healthy [11]. As most farms are located far from microbiology laboratories, the analysis of fresh samples is not always possible, and it may be necessary to refrigerate or freeze bedding samples to avoid undesired microbial proliferation and physical-chemical changes. However, these storage procedures might also impact the reliability and comparability of results [12]. Detailed studies evaluating the impact of sample storage on the reliability of bacteriological procedures on bedding samples are lacking. This study assessed the impact of refrigeration and freezing on bacteriological analysis of SRMS, ADMS, and new sand (**NS**). We considered total bacterial counts as a general indicator of bacterial contamination in the bedding, and Gram-negative bacteria and streptococci to account for the most relevant causal agents of environmental mastitis.

## Materials and methods

### Sample collection and preparation

Unused SRMS, ADMS, and NS were collected in 3 different farms as described previously [13]. SRMS and ADMS were produced in a covered area and used fresh. Sand was brought to the farm, stored under a roof, and used within a few days. For each sample, an aliquot (0.5 kg) was collected in a sterile plastic bag from at least 5 different random locations in the bedding storage area. The samples were kept in a cooler with ice packs and transported directly to the Animal Infectious Diseases Laboratory at the University of Milan (MiLab). Upon arrival, each bedding sample was thoroughly mixed, and 3 aliquots of 150 g each were prepared by taking small sub-samples from at least 3 random locations within the sample. An aliquot was processed immediately (fresh), one was refrigerated at 4 °C for 24 h (+ 4 °C), and one was frozen at − 20 °C for 3 days (-20 °C).

### Bacteriological analysis

At each time point, 25 g of SRMS, ADMS, and NS were transferred to a filter stomacher bag in triplicate, suspended in a 1:10 ratio in Physiological Salt Solution (**PSS**; NaCl 0,9%), and homogenized for 90–120 s at 8 strokes/second in a BagMixer 400 W (INTERSCIENCE, Interlab, Saint Nom la Bretèche, France). Three aliquots of 5 mL were transferred in three sterile tubes and represented the 10^− 1^ dilution triplicates. Serial dilutions were then prepared for each bedding sample, and 100 µL of each triplicate dilution was plated in triplicate onto Cchromogenic (**CHR**) agar (CHROMagar™ ECC, Paris, France) and Edward’s (**EDW**) modified medium (Oxoid, Basingstoke, U.K.), supplemented with 5.0% of bovine blood. CHR allowed the differentiation of Gram-negative bacteria into *Escherichia coli*, coliforms, and other Gram-negative bacteria, while EDW enabled the assessment of streptococci and streptococcus-like organisms (**SSLO**). The plates were incubated at 37 °C for 24 h (CHR) or 48 h (EDW). To determine the total bacterial count **(TBC)**, triplicate dilutions were also seeded in triplicate by pour-plating 1000 µL into molten plate count agar **(PCA)** medium (Microbiol Diagnostic, Cagliari, Italy), followed by incubation at 37 °C for 72 h. In summary, three aliquots were taken from each sample, and each one was analyzed in triplicate on each medium, giving a total of 9 values per microbial class per sample at a single time point. All culture media were prepared and interpreted according to the manufacturers’ instructions. The number of colony-forming units (**CFU**) per mL was assessed as described by Alanis and colleagues (2021), from the dilution plates presenting 2-200 colonies [3]. Bacteriological count values were normalized for the dry matter (**DM**) content and log-transformed [3]. For the DM determination, 10 g of sample were placed for 24 h at 100 ± 5 °C in a dry oven, and the difference between the wet weight and the dry weight was calculated. The results were expressed as Log_10_ CFU/g.

### Statistical analysis

The results were recorded in Excel spreadsheets (Microsoft Office, 2016). Descriptive statistics and non-parametric analysis were performed with IBM SPSS Statistics v. 28.0 (IBM Corp., Armonk, NY, USA). Wilcoxon non-parametric test for paired samples, given the non-normal data distribution (Shapiro-Wilk test), was used. For each bedding material, comparisons between the bedding bacterial counts and the storage conditions effect were carried out. Two comparisons were performed: (a) fresh vs. refrigerated + frozen (comparison between fresh samples results with those obtained under any storage condition), and (b) refrigeration vs. freezing (storage conditions comparisons between each other). Statistical significance was declared when *p* < 0.05. When no colonies were observed, a value of Log_10_ + 1 CFU/g was considered assuming that at least 10 CFU were present in the sample. This arbitrary value was considered due assuming potential losses on each dilution before having the final count [3].

## Results

### Bacteriological results in freshly collected bedding material

The Log_10_ CFU/g average and SD values measured in the three bedding materials either fresh, after refrigeration at 4 °C for 24 h and after freezing at -20 °C for 72 h are detailed in Table 1. In fresh samples, ADMS had the highest total bacterial count of 9.73 (± 0.03), followed by SRMS 9.17 (± 0.05) and NS 6.85 (± 0.03). On the other hand, the higher streptococcal counts were measured in SRMS with 8.37 (± 0.20), followed by ADMS with 5.19 (± 0.11) and NS with 2.96 (± 0.52). Gram-negative bacteria were slightly higher in SRMS 6.43 (± 0.05); in this case, the counts were higher in the inorganic substrate NS 4.96 (± 0.02) than in the organic substrate ADMS 4.82 (± 0.01). When evaluating separately *E. coli*, coliforms, and other Gram-negatives, SRMS exhibited the highest loads for all the three categories. ADMS had higher *E. coli* counts than NS, while NS had higher coliform counts and higher other Gram-negative bacterial counts than ADMS.

**Table 1:** Bacteriological results (mean ± SD) of fresh and cold-stored bedding materials

Bacterial counts (Log_10_ CFU/g)		Separated raw manure solids (SRMS)				Anaerobically digested manure solids (ADMS)				New sand (NS)		
		Fresh^1^	+ 4°C^2^	-20°C^3^		Fresh	+ 4 °C	-20 °C		Fresh	+ 4 °C	-20 °C
Total bacterial count		9.17 (± 0.05)	9.12 (± 0.24)	8.99 (± 0.13)		9.73 (± 0.03)	9.58 (± 0.02)	9.51 (± 0.02)		6.85 (± 0.03)	6.66 (± 0.16)	6.58 (± 0.09)
SSLO^4^		8.37 (± 0.20)	8.32 (± 0.12)	8.21 (± 0.04)		5.19 (± 0.11)	5.17 (± 0.04)	5.21 (± 0.18)		2.96 (± 0.52)	2.81 (± 0.15)	2.79 (± 0.43)
Total Gram-negative		6.43 (± 0.05)	6.43 (± 0.07)	6.17 (± 0.03)		4.82 (± 0.01)	4.80 (± 0.34)	4.37 (± 0.05)		4.96 (± 0.02)	5.03 (± 0.06)	4.24 (± 0.01)
*E. coli*		6.24 (± 0.08)	6.23 (± 0.11)	5.92 (± 0.06)		2.57 (± 0.37)	0.00 (± 0.00)	0.00 (± 0.00)		1.22 (± 10.91)	1.53 (± 10.88)	0.67 (± 8.19)
Coliforms		5.11 (± 0.06)	4.99 (± 0.09)	4.64 (± 0.52)		3.42 (± 1.11)	3.39 (± 0.23)	3.37 (± 0.14)		4.45 (± 0.05)	4.56 (± 0.09)	3.85 (± 0.01)
Other Gram-negative		5.91 (± 0.18)	5.93 (± 0.24)	5.74 (± 0.43)		4.80 (± 0.00)	4.77 (± 0.39)	4.33 (± 0.06)		4.83 (± 0.01)	4.80 (± 0.07)	4.00 (± 0.01)

^1^ Fresh: processed without storage; ^2^ +4°C: refrigerated for 24 h; ^3^-20 °C: frozen for 72 h; ^4^ SSLO: streptococci and streptococcus-like organisms

### Impact of refrigeration and freezing on total bacterial counts

**Fig. 1:**
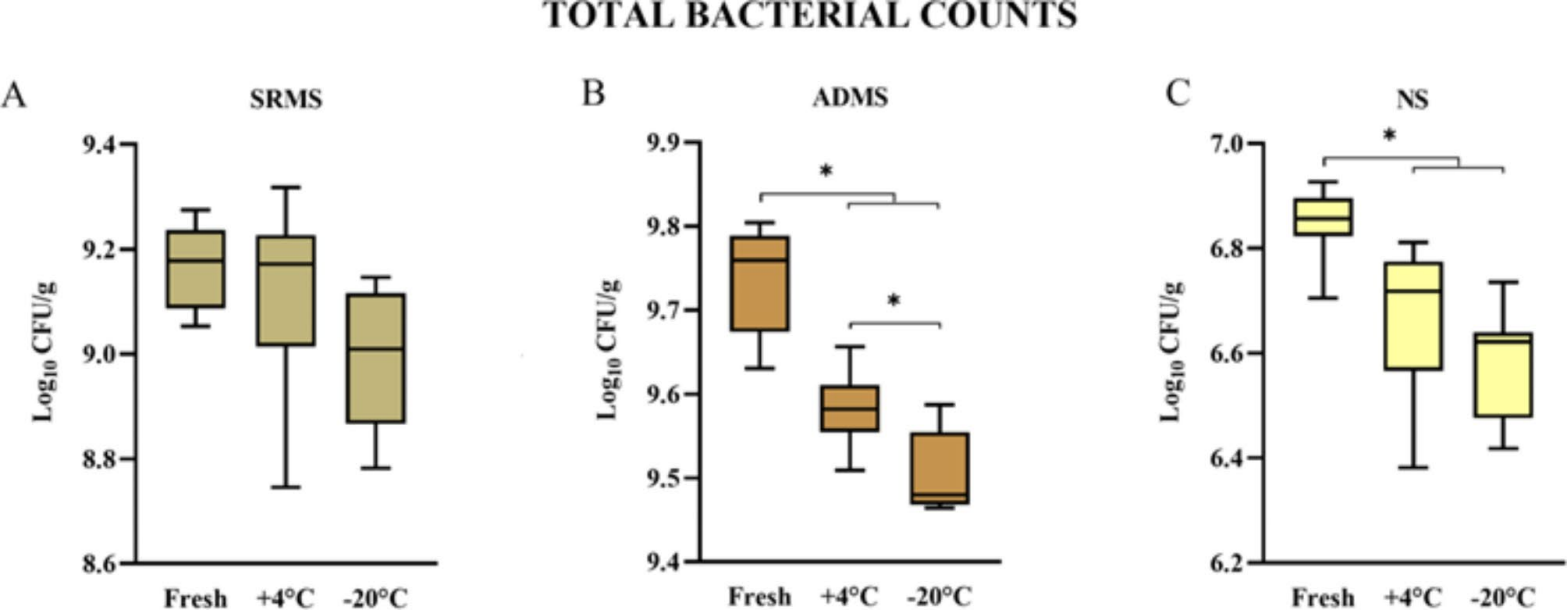
Boxplots illustrating the total bacterial counts in (**A**) separated raw manure solids (SRMS), (**B**) anaerobically digested manure solids (ADMS), and (**C**) new sand (NS) according to the sample storage conditions. Fresh: sample processed immediately; +4 °C: sample refrigerated for 24 h; -20 °C: sample frozen for 72 h. Each boxplot represents the results of the analysis in triplicate of the three subsamples analyzed, for a total of 9 values. *Statistically significant (*p* < 0.05)

The TBC Log_10_ CFU/g average and SD values in the different materials evaluated fresh, upon refrigeration, and upon freezing are detailed in Table 1. The TBC ranged from 8.99 to 9.17 in SRMS, from 9.51 to 9.73 in ADMS, and from 6.58 to 6.85 Log_10_ CFU/g in NS. TBC values were generally reduced by both refrigeration and freezing in all the materials tested, but SRMS (Fig. 1A). The TBC of cold-stored ADMS was significantly lower than fresh ADMS (Fig. 1B); additionally, the TBC of frozen ADMS was also significantly lower than refrigerated ADMS. The TBC was higher in fresh NS compared to both refrigerated and frozen NS (Fig. 1C).

### Impact of refrigeration and freezing on streptococci and streptococcus-like organisms

**Fig. 2:**
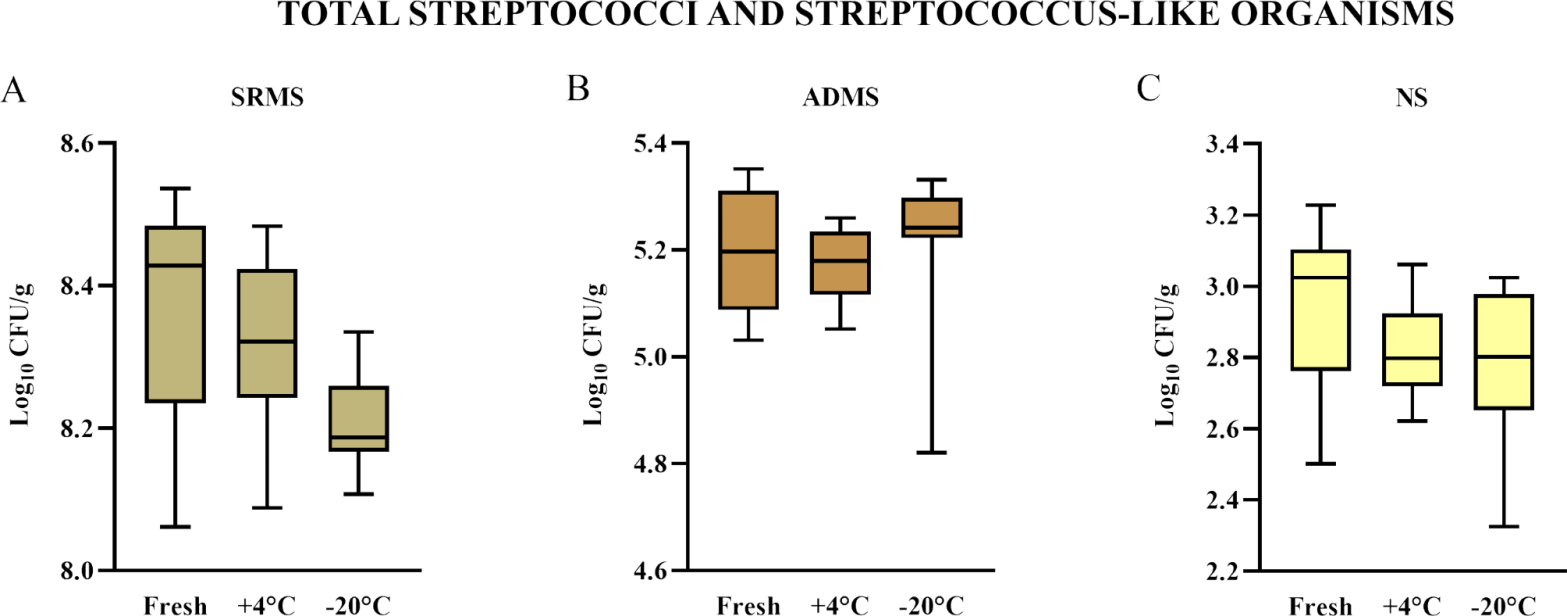
Boxplots illustrating the streptococci and streptococcus-like organism counts in (**A**) separated raw manure solids (SRMS), (**B**) anaerobically digested manure solids (ADMS), and (**C**) new sand (NS), according to the sample storage conditions. Fresh: sample processed immediately; +4 °C: sample refrigerated for 24 h; -20 °C: sample frozen for 72 h. Each boxplot represents the results of the triplicate analysis of three subsamples, for a total of 9 values. *p < 0.05

The total SSLO Log_10_ CFU/g average and SD values in the different materials evaluated fresh, upon refrigeration, and upon freezing are detailed in Table 1. SSLO counts, expressed in Log_10_ CFU/g, ranged from 8.21 to 8.32 in SRMS, from 5.17 to 5.21 in ADMS, and from 2.79 to 2.96 in NS. We did not observe differences of SSLO counts among storage conditions for all the bedding substrates (Fig. 2).

### Impact of refrigeration and freezing on Gram-negative bacteria

The total Gram-negative, coliforms, *E. coli*, and other Gram-negative Log_10_ CFU/g average and SD values in the different materials evaluated fresh, upon refrigeration, and upon freezing are detailed in Table 1.

**Fig. 3:**
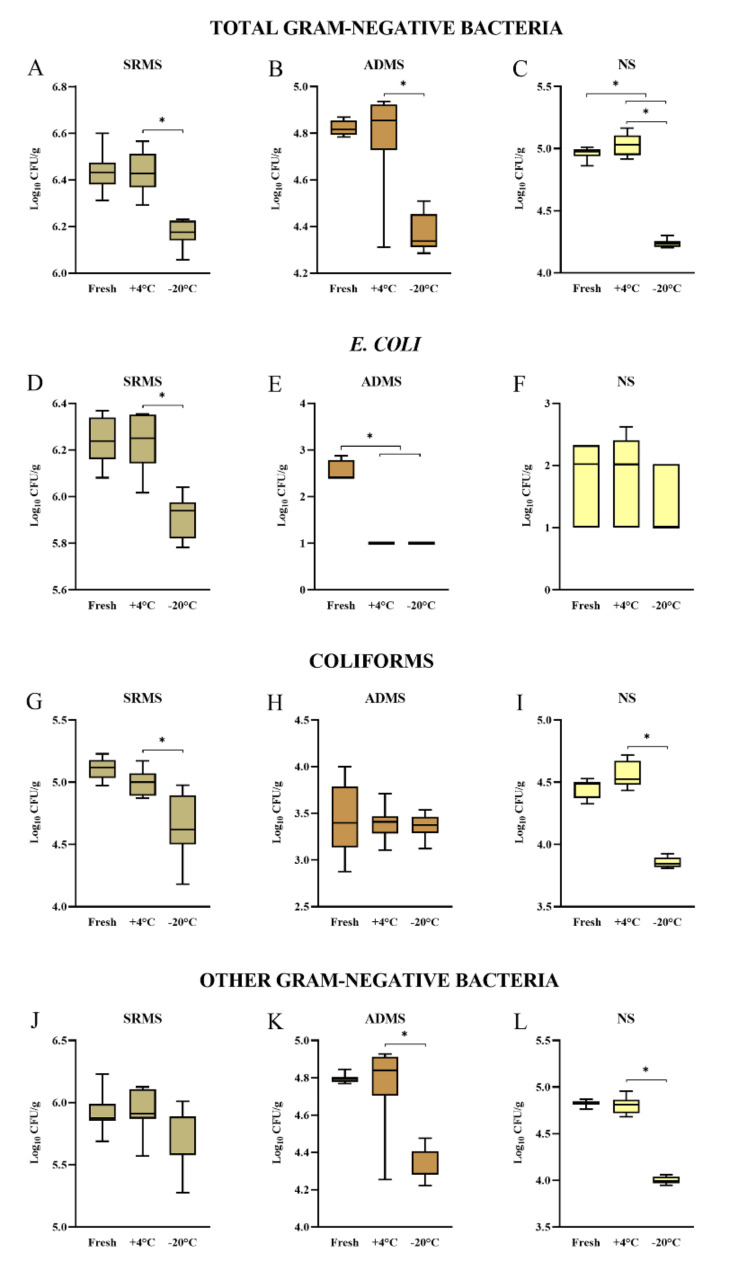
Boxplots illustrating the Gram-negative bacterial counts measured in separated raw manure solids (SRMS), anaerobically digested manure solids (ADMS), and new sand (NS), illustrated according to: total Gram-negative bacteria (**A**,** B**,** C**); E. coli (**D**,** E**,** F**); coliforms (**G**,** H**,** I**); and other Gram-negative bacteria (**J**,** K**,** L**). Fresh: sample processed immediately; +4 °C: sample refrigerated for 24 h; -20 °C: sample frozen for 72 h. Each boxplot represents the results of the triplicate analysis of three subsamples, for a total of 9 values. *p < 0.05

The average total Gram-negative bacterial counts ranged from 6.17 to 6.43, 4.37 to 4.82, and 4.24 to 5.03 Log_10_ CFU/g in SRMS, ADMS, and NS, respectively (Table 1). Total Gram-negative bacterial counts in frozen samples were significantly lower compared to refrigerated samples for all bedding materials (Fig. 3A-C). In fresh NS, total Gram-negative bacteria were also significantly higher than in any cold-stored sample of the same material (Fig. 3C).

The average *E. coli* counts ranged from 5.92 to 6.24, 0 to 2.57, and 0.67 to 1.53 Log_10_ CFU/g in SRMS, ADMS, and NS, respectively (Table 1). *E. coli* counts were significantly lower in frozen than in refrigerated SRMS (Fig. 3D); notably, refrigeration and freezing resulted in no *E. coli* growth in ADMS (Fig. 3E). No differences were observed between sample storage conditions for *E. coli* in NS (Fig. 3F).

The average coliform counts ranged from 4.64 to 5.11, 3.37 to 3.42, and 3.85 to 4.56 Log_10_ CFU/g in SRMS, ADMS, and NS, respectively (Table 1). Coliform counts were significantly lower in frozen than in refrigerated SRMS and NS (Fig. 3G and I); no differences were observed for ADMS (Fig. 3H).

The average counts of other Gram-negative bacteria ranged from 5.74 to 5.93, 4.33 to 4.80, and 4.00 to 4.83 Log_10_ CFU/g in SRMS, ADMS, and NS, respectively. Gram-negative bacteria other than *E. coli* and coliforms were lower in frozen than in refrigerated ADMS and NS (Fig. 3K and L), while no differences were observed between storage conditions for SRMS (Fig. 3J).

## Discussion

To our knowledge, this is the first paper evaluating the influence of storage conditions on the bacteriological and physical-chemical characteristics of SRMS, ADMS, and NS used as cow bedding substrates. The effects of freezing on bedding results were assessed for the first time by Homerosky and Hogan (2015) and published in a conference proceeding [12]. They evaluated the effect of freezing sand, sawdust, and recycled manure for 7, 14, and 21 days, reporting that freezing reduced the Gram-negative bacterial counts in sand and recycled manure.

As expected, we measured higher bacterial counts in organic substrates compared to NS. In our study, the mean TBC of fresh NS (6.85 ± 0.03) and SRMS (9.17 ± 0.05 Log_10_ CFU/g) agreed with other studies [1, 14]. The different counts between substrates can be related to their different compositions, as sand has lower moisture and nutrient contents to support bacterial growth [15]. In our study, the mean TBC for ADMS was 9.37 ± 0.03 Log_10_ CFU/g, which was higher than previously reported [11], probably due to the digester characteristics; the ability of digesters to reduce the presence of pathogens depends on many factors, including the temperature and the feedstock composition [16].

The Gram-negative bacterial counts in fresh samples of SRMS, ADMS, and NS were in line with other studies [14, 17, 18]. The bacterial loads observed for SSLO were also in line with recent studies [18–20], although Rowbotham and Ruegg (2016) found a higher number of streptococci and streptococci-like organisms in unused sand, recycled sand, and manure solids [21].

Overall, we observed that refrigeration at 4 °C for 24 h affected bacterial counts less than freezing at -20 °C for 3 days. Gram-negative bacteria counts were lower in frozen than refrigerated samples of all substrates tested, in agreement with Homerosky and Hogan (2015) [12]. Specifically, a significantly higher reduction of coliforms in SRMS and NS was observed after freezing compared to refrigeration, as well as for *E. coli* in SRMS. In ADMS, *E. coli* became undetectable after freezing, in agreement with other studies [22, 23]. Masters and colleagues (2015) observed a reduction of *E. coli* in fecal samples preserved at -20° for 30 days [22], and the same trend was observed by Schukken and colleagues (1989) in milk samples [23]. The formation of intracellular and extracellular ice crystals might be responsible for cellular damage. Adding to this, freezing of extracellular water can lead to an increase in mineral concentrations, causing a shrinking of the cell with possible membrane lesions [23, 24].

Concerning SSLO, no difference was observed between fresh and cold-stored samples, in agreement with Homerosky and Hogan (2015) [12]. This results also corroborates the findings of Wang and colleagues (2004), who reported no significant reductions in fecal streptococci counts at different temperatures or humidity rates [25]. Given their cell wall structure, Gram-positive bacteria might be less susceptible to physical damage due to freezing.

Our results have practical implications for farmers and veterinarians submitting bedding samples for microbiological analysis. Cold storage methods may lead to underestimate microbial content compared to fresh samples, so results should be interpreted with caution. Fresh samples are preferable for accurate microbial counts, but if this is not possible, refrigeration is better than freezing. Different targets for acceptable microbial counts may be required for cold-stored samples.

Finally, we observed a large variability within bedding samples, despite using standardized operating procedures. A limitation to consider is the variability of bacterial populations in relation to farm management practices and different types of separators and digestors. These variables can have a significant effect on the microbial composition of RMS. Differences in feedstock, temperature and processing techniques in anaerobic digesters can affect the survival and abundance of bacteria in ADMS. Similarly, variations in mechanical separation methods for SRMS can result in different levels of microbial contamination in the final bedding material. We should also consider the variable definitions of recycled manure materials found in the literature. These are often considered together as recycled manure solids (using the acronym RMS) or dried manure solids (using the acronym DMS) without specific reference to how these were obtained or treated, making it difficult to retrieve and compare previously obtained data. These aspects should be considered in further research.

## Conclusions

Our study underlines the importance of adopting appropriate storage procedures of samples before microbiological analysis of bedding materials for dairy cows. Specifically, we highlight that cold storage can significantly reduce bacteriological counts, compared to fresh bedding samples, which should be preferred over cold-stored samples. When the use of fresh samples is not possible for logistic reasons, refrigeration should be preferred over freezing, while acknowledging that this might underestimate the actual microbial content. Microbiological counts obtained from refrigerated or frozen bedding samples should be interpreted carefully. Our results can guide technicians and researchers interested in assessing the microbiological characteristics of bedding materials for dairy cows, when dealing with refrigerated or frozen samples.

## Data Availability

Not applicable.
